# Food Allergies and Parasites in Children

**DOI:** 10.3390/foods12132465

**Published:** 2023-06-23

**Authors:** Kacper Packi, Alicja Rudek, Joanna Matysiak, Sylwia Klimczak, Eliza Matuszewska, Natalia Rzetecka, Jan Matysiak

**Affiliations:** 1 Department of Inorganic and Analytical Chemistry, Poznan University of Medical Sciences, 60-806 Poznan, Poland; eliza.matuszewska@ump.edu.pl (E.M.); natalia.rzetecka@student.ump.edu.pl (N.R.); jmatysiak@ump.edu.pl (J.M.); 2 AllerGen Center of Personalized Medicine, 97-300 Piotrkow Trybunalski, Poland; alicjarudek@o2.pl (A.R.); sylwia.klimczak2@stud.umed.lodz.pl (S.K.); 3 Faculty of Health Sciences, Calisia University-Kalisz, 62-800 Kalisz, Poland; jkamatysiak@gmail.com; 4 Department of Nucleic Acid Biochemistry, Medical University of Lodz, 92-213 Lodz, Poland

**Keywords:** food allergies, parasites, *Anisakis simplex*, tropomyosin

## Abstract

The dynamically growing incidence of food allergies forces the scientific community to develop new methods for their diagnosis, differentiation, and effective treatment. Parasitoses appear much less frequently in the scientific literature, as well as among the presumed causes of numerous conditions. The similarity of inflammatory mechanisms in allergies and parasitosis necessitates a revision of current diagnostic standards. A lack of specificity and the coincidence of symptoms at an early stage of disease can lead to misdiagnosis. In this paper, we attempted to perform a comparative analysis of the similarities and differences in symptoms for these two types of diseases. We described the molecular mechanisms and metabolic pathways of food allergy and parasitosis. We presented the available research methods and directions of ongoing studies aimed at implementing precise medical techniques for differential diagnosis. We discussed the allergenic properties of certain parasite proteins, using the example of myofibrillar tropomyosins from the nematode *Anisakis simplex*. The literature in the fields of allergology and parasitology leads to the conclusion that it is reasonable to run parallel allergological and parasitological diagnostics in patients with non-specific symptoms. This approach will facilitate accurate and early diagnosis and implementation of effective therapy.

## 1. Introduction

The modern world fosters the spread of numerous pathogens via integration and thus an increased migration of people. The thriving economies alter the natural human, animal, and microbial ecosystems. Changing environmental conditions pose a great threat to human immunity and health. Numerous chemicals present in the environment in which humans live enter their bodies via various routes, causing, inter alia, allergic diseases [1]. The anthropogenic impact on the environment has a direct effect on human health. Industrialization and changes taking place in the world result in a sharp increase in the number of so-called civilization diseases, which include allergic diseases [2].

One reason for the onset of food allergies may be the child’s immature intestinal barrier. This impairs the immune mechanisms and disturbs the Th1:Th2 lymphocyte ratio [3]. An increased number of Th2 lymphocytes, on the other hand, results in reduced food tolerance and immune response to infection [3]. Gut microbiome depletion not only promotes the development of allergic diseases but can also cause autoimmune disorders and parasitic infections [4]. Increased, prolonged intestinal barrier permeability, along with individual genetic predispositions, can trigger an immune response [4]. According to the literature, some parasitoses cause allergy-like symptoms [5]. For this reason, non-specific symptoms of infestation are often attributed to other disorders, including those of allergic origin. Numerous authors of scientific papers identify parasitoses and allergy diseases as a worldwide problem [6,7,8]. The purpose of this paper is to emphasize the importance of including parasite allergens in the initial diagnosis of allergic diseases. It is deemed necessary to introduce parasitological testing in the diagnosis of allergies in cases where there is a positive reaction to parasite allergens. We attempt to analyze and compare the similarity of symptoms and metabolic pathways between food allergies and parasitic infestations, as well as the correlation of underlying pathogenic mechanisms. This review is based on original and review papers published between 2010 and 2023, presenting the current state of the art in the field of both diseases.

## 2. Epidemiology of Food Allergy

According to data from the European Federation of Allergy and Airways Diseases Patients’ Associations (EFA) the problem of food allergy (FA) affects 17 million people in Europe, 3.5 millions of whom are younger than 25 years old [9]. Nearly 60% of FA cases in adults and children co-occur with respiratory allergies [10]. Numerous scientific reports indicate that immune system failure underlies these conditions, which is not only determined genetically but also by lifestyle and the environment. Allergic diseases affect as much as 30–40% of the world population, with FA taking a special position among other types of allergies. It occurs both in adults (1–2%) and, especially, in the youngest children, i.e., infants and children under three years of age (6–8%) [11]. Among the most commonly described food allergens, there are cow’s milk, eggs, fish, nuts, wheat, and soy. For example, cow’s milk contains about 20–30 proteins that can be allergenic [12]. Of those, the most often described as allergy triggers are casein and α- and β-lactoglobulins [13,14,15].

The prevalence of allergic symptoms varies depending on the age and sex of patients [16]. The prevalence of allergy is estimated to be about 1.4–2% in the adult population, with twice as many symptoms of food hypersensitivity found in women. In children, FA occurs in about 5–8% of the population [16]. It is believed that the prevalence of FA is high and constantly increasing both in Poland and throughout Europe [17]. In Poland, more than 20% of the pediatric population is affected. Moreover, in recent years, the incidence of FA in children under the age of five has doubled [13]. The highest frequency of FAs in children under the age of five is due to the fact that a significant proportion of affected children diagnosed with FA over time begin to tolerate allergenic products [18]. This usually takes place within 1–3 years of diagnosis and appropriate treatment. Kaczmarski et al., in the analysis of the natural history of allergy to cow’s milk proteins conducted in a group of 291 children aged 2 to 14 years, found that 200 of them (72.9%) gained tolerance at the average age of five years after using an elimination diet [19]. Pyziak et al. observed 115 children with IgE-mediated allergy to cow’s milk proteins diagnosed during the first three years of life, for a minimum of five years from diagnosis [20]. It was found that 87.9% of them acquired tolerance, and the frequency of acquiring tolerance increased with the age of the children.

A wide array of external factors affects the development of allergy; however, genetic predisposition also plays a role. Several genes responsible for “allergy predisposition” have been identified so far. They are located on chromosomes 5 and 11, and the interplay between these genes leads to the so-called atopic phenotype. The genes located on chromosome 5q31-33 control the production of IgE and also encode interleukin (IL)-3, IL-4, IL-5, IL-13, and granulocyte-macrophage colony-stimulating factor (GM-CSF), which are responsible for maintaining the chronic inflammatory allergic response [21]. The incidence of allergic diseases also varies between country regions [22]. The place of residence is also crucial; for example, in rural areas, fewer cases are reported. Children growing up in the natural ecosystems of the countryside are exposed to an increased number of microorganisms, which results in a faster development of immune mechanisms. Changing environmental conditions and lifestyles are therefore primarily responsible for the increased incidence of allergic diseases [23].

## 3. Epidemiology of Parasitic Diseases

It is estimated that more than two billion people worldwide are infected with intestinal parasites, and five billion live in areas with a high risk of infection with invasive pathogens [24].

The most common intestinal parasite in the human population worldwide is the common roundworm (*Ascaris lumbricoides*). It is the causative agent of ascariasis, an invasive disease affecting, according to various estimates, 800 to 1200 million people [21,22]. Giardiasis (caused by *Giardia duodenalis*) is another important parasitosis. According to the literature, the disease is diagnosed based on symptoms in about 280 million people per year and is most common in developing countries. It is estimated that in developed countries, giardiasis occurs in 2–5% of the population, while in developing countries, 20–30% of the population is infected [25]. In Poland, according to the National Institute of Public Health–National Institute of Hygiene, in 2012, there were 1655 cases, in 2013, there were as many as 1880, and in 2019, there were 785 infected people registered [26]. However, these data are not complete, and no reliable statistics were collected during the COVID-19 pandemic. Giardiasis, along with cryptosporidiosis (caused by protozoan parasites from the genus *Cryprosporidium*), was included in the World Health Organization Neglected Diseases Initiative in 2004 [27]. Some parasites are geohelminths (i.e., soil-transmitted helminths), meaning they release eggs that become infectious in the soil. Studies on the contamination of natural human habitats directly indicate the degree of risk of a parasitic infection. Among all geohelminths, the eggs of common roundworms are most often found in the soil and sand of public green areas. Geohelminths are also found in samples taken in rural areas. The self-purification of soil is slower in rural yards, and human companion animals are much less likely to be dewormed [28]. The soil analysis showed that the determining factor to classify the soil irrigated with wastewater as contaminated was the presence of *A. lumbricoides* eggs [29]. Ascariasis is most commonly recorded in sub-Saharan Africa, China, Southeast Asia, and the Americas [28]. The analyses performed in Mexico revealed the presence of *A. lumbricoides* in 77% of school-aged children [30]. In Ivory Coast, on the other hand, about 36% of school-aged children were found infected with geohelminths. In Cameroon, infestations with this parasite are the most common cause of parasitic diseases [31]. Fertilization of the soil with feces is to date practiced in many villages. Pig roundworm (*Ascaris suum*) is genetically related to the common roundworm (*A. lumbricoides)* and is considered a potentially zoonotic parasite. Cases of human infection with *A. suum* have been described and confirmed by molecular tests [32].

Most parasitic diseases are distributed worldwide [33], and infections with various parasites “immigrating” from many parts of the world occur due to large-scale human migrations. A significant increase in the number of people traveling for professional and touristic reasons to developing countries, as well as an influx of immigrants to Poland, is likely to result in an increased prevalence of parasitic infections and a rise in disease rates in the Polish population over the next few years [34].

## 4. Common Symptoms of Food Allergy and Parasitic Infestation

**Figure 1:**
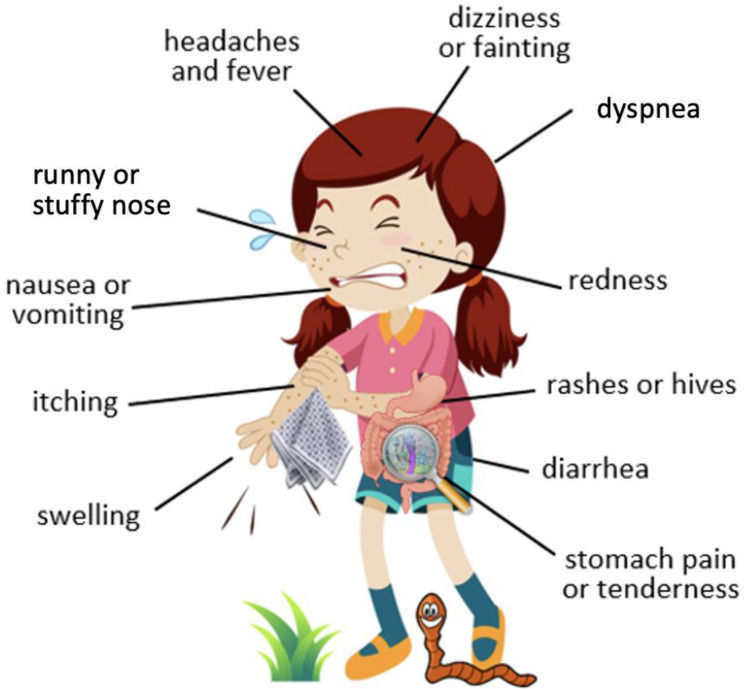
Symptoms common to food allergy and parasitic infestation.

The immune processes and reactions that take place during the body’s response to parasitic invasion and allergic disease may have similar external symptoms [5]. Numerous papers describe analogous symptoms in both types of diseases. The similarity is primarily due to the activation of the same body’s defense mechanisms during contact with foreign proteins [5]. The reactions and their severity depend on multiple factors. If the invasion with a parasite occurs accidentally in a region that is not endemic to it, usually, the infected organism reacts very violently. Hyperergic reactions, such as Löffler’s syndrome or eosinophilic pneumonia, can then occur, requiring rapid, targeted therapy with systemic corticosteroids [35]. Allergic reactions of the immediate type follow a similar course [5]. Symptoms of acute extrinsic allergic alveolitis resemble those of an acute respiratory infection. These include shortness of breath, elevated temperature, chills, malaise, joint pains, and cough [36]. An acute reaction to parasites can be similar to an acute allergic reaction to inhalants or food allergens [35]. Thus, precise diagnostics and an accurate diagnosis are crucial. The body’s reactions to helminths and allergies are also consistent. Intestinal nematodes cause symptoms related to gastrointestinal dysfunction, such as diarrhea and abdominal pain, as well as general weakness of the body [37]. The life cycle of some parasites involves larvae that seek a convenient place to develop in the host body. The migration of the larvae is accompanied by symptoms of allergy, that is, papular, itchy rashes, hives, and less commonly conjunctivitis, wheezing, shortness of breath, and occasionally hemoptysis [38]. Almost identical ailments are observed in patients allergic to certain foods. FA can manifest as skin lesions, e.g., rash, hives, and angioedema [39]. In asthma, on the other hand, an undesirable symptom can be bronchial hyperresponsiveness, leading to attacks of wheezing, shortness of breath, a tight chest, and coughing [36]. Other distinctive symptoms of some allergic diseases are a skin rash or a strenuous cough. In the course of ascariasis, similar skin lesions and/or a cough that resembles allergy symptoms are reported. The larvae, released in the gastrointestinal tract of the host, gradually mature and migrate through the portal system and lungs to the respiratory tract. The presence of larvae in the laryngeal region causes coughing with their expectoration [40]. Figure 1 presents symptoms common to allergy and parasitosis manifested during the course of both types of diseases.

Parasites can modify immune events and favor those that allow them to survive [41]. The consequence of the parasite’s defensive actions to survive in the host body can be variable alarm signals resembling allergic reaction or parasitic invasion. This phenomenon is associated with antigenic variability—the immune response is modified because subsequent stages of the parasite’s life cycle may have different antigens [42]. The body’s reaction to the metabolic products the roundworms secrete is the cause of other symptoms of helminthiasis. Parasite metabolites often cause more serious disorders in the human body than the presence of the parasite itself [43]. External, non-specific symptoms often affect toddlers and are reported by parents when visiting doctors’ offices [41,42]. Children are a large group of allergic patients, and thus, more often than not, the symptoms they experience are associated with allergic diseases [41,42].

## 5. Molecular Mechanisms and Immune Response Pathways in Food Allergies (Mainly IgE-Mediated) and Parasitic Infestations

**Figure 2:**
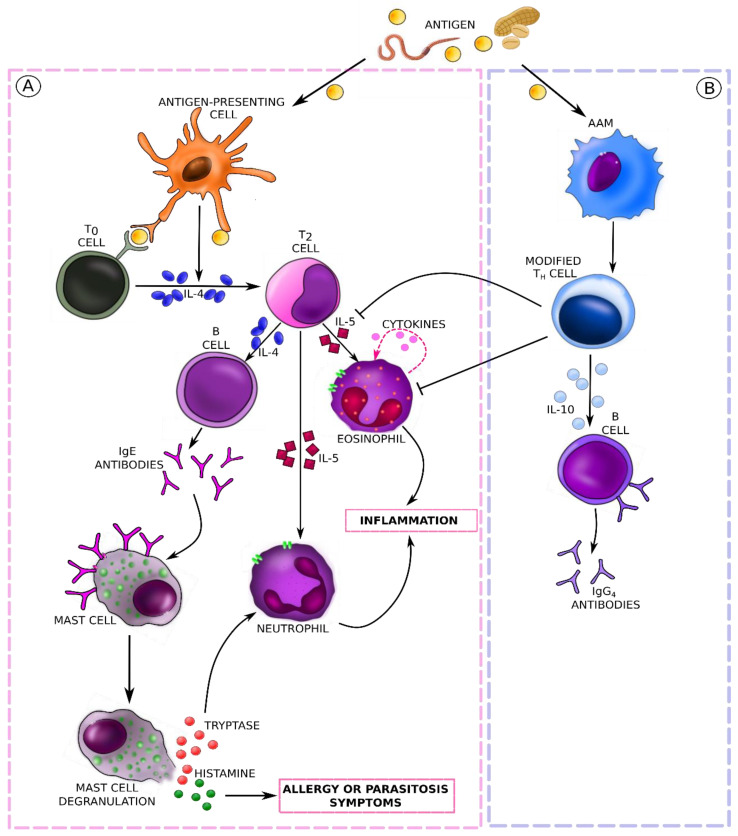
Mechanism of immune response in allergic and parasitic diseases. (**A**) Immediate response: contact with a food allergen or a parasite triggers an immune response. APC cells presenting the antigen to Th0 lymphocytes trigger the cellular response of the Th2 pathway. The cytokines produced by Th2 (i.e., IL-4 and IL-5) have certain roles. First, IL-4 stimulates the synthesis of the IgE antibodies causing mast cell degranulation. On the other hand, IL-5 is a signal for eosinophil and neutrophil cell influx, triggering the inflammatory response and manifestation of symptoms. (**B**) Chronic response: long-term presence of the parasite can lead to modification of Th lymphocytes, which release IL-10 which stimulates B lymphocytes to produce IgG4 antibodies. APC: antigen-presenting cells.

Allergy and parasitic infections initiate identical immune responses in the initial phase of the disease. Parasitic infestation is often accompanied by immediate-type symptoms similarly to the reaction to the body’s contact with an allergen [44,45]. In both cases, we speak of hypersensitivity to the stimulus and observe an inflammatory process of a similar nature. In the pathogenesis of both diseases, the immune system, involved in the production of antibodies of various classes, plays a primary role. This is a response to foreign proteins, that is, allergens or tissue proteins and metabolites of the parasite [46]. Once an unknown protein enters the body, the immune system activates the first line of defense. This process involves cells such as T and B lymphocytes, regulatory T cells, eosinophils, neutrophils, mast cells, basophils, etc. [47]. T cells are essential in the regulation of the immune response. Depending on the activation of a certain subpopulation of T helper cells (Th), a cell-mediated or humoral response is triggered. Th1 cells participate in the induction of the cell-mediated type of response, while Th2 cells mediate a humoral immune response. These subpopulations differ in the profile of secreted cytokines [41]. Both the course of allergic diseases and the progress of parasitoses are characterized by a higher response from Th2 cells with increased serum levels of IL-4, IL-5, and IL-13, as well as eosinophilia and elevated IgE levels [42]. Th2 cells develop from Th0 lymphocytes, and the process is supported by, inter alia, the action of IL-4, which is described as the primary cytokine in the pathomechanism of allergic diseases. Basophils are thought to be an important source of IL-4 in the early stages of sensitization to allergens and parasite antigens [48]. Parasites are the strongest known inducers of Th2-type immune response [49], but the presentation of allergens on the surface of mature dendritic cells also stimulates the proliferation of this subpopulation. The activity of Th2 cells is mainly associated with allergic reactions: the cells, through secreted cytokines, stimulate IgE antibody production and have the ability to activate eosinophils, basophils, and mast cells [50]. Stimulation of Th2 cells leads to the secretion of IgE and IgG antibodies by plasma cells and induces eosinophilia [48]. First, Th2 lymphocytes release cytokines that stimulate B lymphocytes to differentiate into plasma cells, and then, these cells trigger the production of IgE antibodies against specific antigens. The production of large amounts of IgE results from the formation of long-lived plasma cells and memory cells [48]. As the defense and inflammatory processes develop in the course of allergic and parasitic diseases, the immune pathways become specific to each of them. The pathological mechanism of the immune response after contact with a food allergen or a parasite protein is shown in Figure 2.

The mechanism of allergy is a complex and multi-step process. According to the current recommendations of the European Academy of Allergology and Clinical Immunology (EAACI) published in 2001, allergic reactions are classified as IgE-mediated (type I reactions according to the Gell and Coombs classification), non-IgE-mediated (type II-IV reactions according to the Gell and Coombs classification), and other reactions proceeding with the activation of immune mechanisms [51]. According to literature reports, FAs are most often related to type I hypersensitivity, i.e., IgE-mediated reactions. This is also confirmed by recent studies on milk protein allergy. In Poland and Europe, IgE-mediated allergy to cow’s milk proteins is much more common than non-IgE-mediated allergy and is also characterized by a milder clinical presentation [52]. An IgE-mediated reaction is one whose underlying pathomechanism is the interaction of an antigen or allergen with IgE antibodies. IgE antibodies bind to surface membrane receptors on mast cells, i.e., immunocompetent effector cells, and then bind the specific allergen [53]. There are two IgE-binding receptors: a high-affinity receptor (FcεRI) and a low-affinity receptor (FcεRII or CD23). FcεRI is a tetrameric receptor present on mast cells and basophils (αβγ2) and a trimeric receptor (αγ2) on antigen-presenting cells such as monocytes, Langerhans cells, and dendritic cells in the peripheral blood [54]. When a mast cell encounters an allergen or antigen, it releases inflammatory reaction mediators from storage granules. Stimulation of the mast cells, which secrete a variety of potent substances (primarily histamine and tryptase), results in the development of clinical symptoms of allergy. The mast cells are present in the so-called shock organs, i.e., in the gastrointestinal tract, nasal and ocular mucosa, skin, and bronchi. They play an important role in defense against parasites and microbes by inducing a local inflammatory response [35].

Clinical symptoms, as well as the course of the allergic response itself, can be divided into two phases: immediate and late [52,53]. The immediate phase is mainly associated with the release of effector substances from the mast cells, which can present clinically as itching, sneezing, a runny nose, bronchospasms, or hives. The late-phase response, however, is associated with an influx of residual inflammatory cells. It appears 6 to 12 h after the contact with the allergen, lasts longer, and is much more difficult to treat. Symptoms of this phase can include, for example, nasal congestion or bronchospasms [52,53]. It is important to remember that both acute and delayed reactions, as well as IgE-mediated and non-IgE-mediated reactions, can occur in the same patient [55]. In addition, the same symptoms can be associated with either IgE-mediated or non-IgE-mediated reactions, especially with gastrointestinal manifestations [56]. Eosinophils (acidophils) are also among the inflammatory cells activated during an allergic reaction. The granulocytes contain a number of proteins with cytotoxic properties in their granules, such as major basic protein (MBP; it accounts for more than 50% of their composition), eosinophil peroxidase (EPX), eosinophil cationic protein (ECP), and eosinophil-derived neurotoxin (EDN). These proteins play an important role in the elimination of multicellular parasites, including helminths. Eosinophils accumulate in tissues involved in allergic inflammation, where, in the presence of allergens, they can participate in tissue destruction [57]. Laboratory testing indicates significant eosinophilia in both allergic diseases and some parasitic infestations. Proinflammatory cytokines, such as IL-1β, IL-2, IL-12, and tumor necrosis factor alpha (TNF-α), are involved in regulating the infiltration of eosinophils into inflamed sites [41]. Contact of the allergen with the body can occur through the mucous membranes of the gastrointestinal tract (food allergens), respiratory system (inhalant allergens, mainly plant pollen), skin (contact allergens), and conjunctiva [58]. The mucous membrane of the gastrointestinal tract is permeable to protein and carbohydrate molecules that enter the intestinal lumen. Their binding or adsorption by the intestinal epithelium occurs, and they are then released into the intercellular space via lysosomes. Some food allergens can penetrate the intercellular space in unchanged form directly through the intercellular junctions [59].

Increased mucosal permeability is also observed in the course of certain parasitic infestations [59]. It is also associated with the immaturity of the gastrointestinal tract, which explains the high prevalence of FAs in the younger age groups [60]. The consequence of increased mucosal permeability is the passage of unwanted particles of undigested food, allergens, microorganisms, intestinal parasites, and toxins into the blood. The state of chronic overloading of the immune system with foreign antigens directly decreases immunity, which in turn drives allergy or the rampant development of viral, bacterial, and parasitic infections. During parasitosis, the order and course of metabolic pathways can vary. Parasites can modulate the order and course of the reactions due to the variable antigenic surface coat of their developmental forms [61]. This ability facilitates their survival and development in the host body. The exchange of surface antigens in, inter alia, flukes and tapeworms provides an effective defense against the specific antibodies present in the gastrointestinal tract [61]. The immune response stems from the induction of subsequent reactions by different developmental stages of the parasite [62]. The host defense system, however, modulates these reactions so that they lead to the elimination of the parasite. Nevertheless, the sequence of events in the initial stage of a parasitic infestation resembles the induction of an allergic reaction. Parasitic infection induces a wide array of immune processes, such as eosinophilia, mastocytosis, alternative activation of macrophages, maturation of B lymphocytes, and production of antibodies [60,61] (mainly of IgE and IgG1 classes), as well as activation of Th2 lymphocytes producing IL-4, IL-5, and IL-13. Depending on the type and species of the invading parasite, the body’s response may differ. The allergic immune response causes the elimination of intestinal nematodes, while the cellular response has been attributed a defensive function directed mainly against the larvae of flukes and tapeworms. The response of the host organism to infestation does not always resemble a purely defensive response. Defensive and pathological responses are usually intertwined and overlap [41]. Parasites are masters at modulating the immune response of an infected organism. They employ a variety of strategies to thrive and multiply. One such strategic action is their ability to induce immunosuppression in the host. Inhibiting the production of antibodies and immune cells allows the parasites to reach their target organs. In addition, their adaptive capabilities allow them to exploit the host’s metabolic signals for their own development [63]. It has been shown that during parasitic infection, natural regulatory mechanisms are induced that safeguard the host against excessive inflammation or autoimmune reactions. For example, regulatory T cells (Treg) are responsible for tolerance to autoantigens and for limiting excessive responses to foreign particles [64]. Parasites secrete a number of specific metabolites that directly affect the host. They can affect T cells and alter their capacity for proliferation or apoptosis [65], which affects the balance of the Th1/Th2/Treg response. In addition, some helminths induce apoptosis (i.e., the so-called programmed death) of various immune effector cell populations [66]. Some helminths affect the level of antibody production by switching the expression of genes encoding IgE antibodies to IgG4 antibodies. The latter, unlike IgE, are not involved in the induction of parasite elimination, and their presence indicates the development of allergen tolerance [67]. Parasite presence wreaks havoc in the host’s body and disrupts its internal homeostasis. The imbalance can lead to the development of chronic inflammation accompanying numerous civilization diseases, including autoimmune conditions and allergies [68]. However, studies show that this antagonistic interaction between two species does not always exclusively harm the host. As a result of the evolutionarily long-lasting “cooperation” between the parasite and its host, in addition to the pathological effects of infection, helminths can be observed to influence the maintenance of immune homeostasis [41]. Studies conducted in recent years on the inhibition of inflammatory response by some intestinal nematodes may prove some types of parasitic infection to be an excellent alternative in the treatment of autoimmune diseases and allergies [68].

## 6. Diagnosis of Food Allergy

Over the years, allergy diagnostic standards have changed [69], starting from careful observation of typical clinical symptoms, through the use of skin prick tests (Ch. Blackely), to the measurement of serum IgE levels (Kimishige and Teruko Ishizaka) [69]. Still, the basis for a correct allergy diagnosis is a detailed and reliable interview that enables the assessment of the correlation between symptoms and an allergen exposure [69]. Diagnosing an FA is more problematic. Patients with FAs form a heterogeneous group, differing in the causes of their symptoms, pathogenesis, and clinical picture [70]. An FA is mainly suspected based on an interview [70], only some symptoms can be objectively assessed during physical examination. In vitro and in vivo tests verify the IgE-mediated mechanism of hypersensitivity, but they are not fully matched [70]; there are no methods for the laboratory verification of a non-IgE-mediated allergy [70]. The sensitivity and specificity of sIgE detection methods vary, and they are often unsatisfactory [70]. There is also a high risk of false-negative and false-positive results. Due to processing, the extracts used in the tests can differ from the natural, original substrates [68,69]. Moreover, food consumed by a patient can, during ingestion, become a source of new antigens that induce the synthesis of IgE-specific antibodies. The gold standard in diagnosis is a double-blind, placebo-controlled provocation test [71]. It has been used successfully for many years, and it helps to confirm whether a patient is allergic to a given food in the case of a non-IgE-mediated allergy when other methods cannot be used [71]. The availability of in vivo diagnostics is limited. It can be health- and life-threatening, and the test conditions differ from the conditions of natural exposure. Sometimes, allergy symptoms appear only a few hours or days after consumption, which is why an elimination diet is required [69]. Diagnosis is hampered by the variety of foods consumed, the variability of their allergenic properties, and the presence of cross-allergies [72].

The allergological interview requires detailed information from the patient on all topics that can be relevant to a possible allergic disease [46]. Anamnesis should assess the type of food or its components that are the probable cause of the symptoms, the amount of food consumed, the time from consumption to the onset of hypersensitivity reaction, conducive factors (i.e., exercise, drugs, or stimulants), and the symptoms and consequences of the allergic reaction [73]. Based on the results of this examination, the allergist decides on further diagnostic procedures. The physician assesses the probability of an allergy and orders relevant allergic tests. Laboratory/clinical tests, i.e., skin prick tests (SPT), food challenges, and in vitro tests for specific class E immunoglobulins (sIgE) are used as additional tools to verify the initial diagnosis [71,72,73].

Despite significant advances in allergology, a properly conducted, double-blind, placebo-controlled food challenge (DBPCFC) remains the gold standard of food allergy diagnosis [71]. Although the DBPCFC is the most specific test, it is time-consuming and may cause adverse allergic reactions, which makes it rarely used in clinical practice [71]. The DBPCFC test is generally conducted by specialists under the supervision of a physician, and it involves the patient ingesting increasing amounts of potentially allergenic food [74]. The symptoms of hypersensitivity are then observed. Based on the DBPCFC results, an elimination diet, devoid of allergenic ingredients, is developed. The disappearance of symptoms and their reappearance after consumption of the tested ingredient confirm an FA. A food challenge may have different forms, for example, an open challenge, a single-blind challenge, and a double-blind, placebo-controlled challenge. In an open challenge, both the physician and the patient know what type of food the patient consumes during the test. In a single-blind challenge, the physician knows the type of food consumed by the patient, but the patient does not know it. In a double-blind, placebo-controlled challenge, neither the physician nor the patient knows what type of food the patient consumes.

A breakthrough in the history of allergy diagnostics is the development of molecular diagnostics related to genetic engineering [67]. Molecular diagnostics, also called component-resolved diagnostics (CRD), is used to confirm the presence and assess the concentration of IgE antibodies specific to allergen molecules in the serum [75]. The first results on molecular methods in allergology were published by R. Valent at the end of the 20th century [73,74]. Assuming that any protein-containing particle can be an allergen, researchers started to test allergic reactions to specific proteins and not to the food containing them. To date, the main allergen families of key importance in allergology have been discovered and characterized [76]. Undoubtedly, the methods of genetic engineering have greatly contributed to the development of allergology. Knowledge of the specific allergenic protein, and not only the source of allergy, brings about a number of benefits [76]. The methods of molecular diagnostics significantly increase the specificity of the determination, as they offer the possibility of assessing the primary allergy and improving diagnostic sensitivity, especially when clinically significant allergens are scarce or absent in the extract [73]. The CRD assesses the probability of cross-reactions, predicts the severity of a systemic allergic reaction, and determines and specifies the indications for allergen immunotherapy [73]. The possibility to detect specific antibodies against major allergenic molecules facilitates the identification of the root cause of clinical symptoms [73]. Identification of the primary allergy is the basis for targeted prophylaxis and optimal therapy [73]. Basic diagnostic methods, i.e., skin prick tests and asIgE against allergen extracts, do not allow for assessing the presence of IgE specific for individual allergens (proteins) contained in source extracts, which entails a risk of false-positive or false-negative results [68]. The reasons for this include the different content of individual allergen molecules in the source extracts, their clinical significance, and the prevalence of cross-reactions. The cross-reactions often result in the misinterpretation of the allergic test results. Identification of the molecules contained in the specific source allergens and knowledge of their potential cross-reactions are indispensable for a correct diagnosis. A special case is patients at a risk of anaphylaxis due to food allergens, where the correct interpretation of the test result and the possibility of detecting the anaphylactogenic allergen offered by molecular diagnostics may save their lives [68]. CRD is also extremely helpful in establishing indications for allergen immunotherapy [77]. Advanced nanotechnology used to detect serum antibodies against major and minor allergens enables one to introduce proper immunotherapy, establish the composition of an allergen vaccine, and monitor the therapy effectiveness [77]. Individually adjusted therapy and good treatment results achieved thanks to CRD seem to confirm the value of the new diagnostic methods and encourage the researchers to broaden their knowledge in this field [75,76].

**Figure 3:**
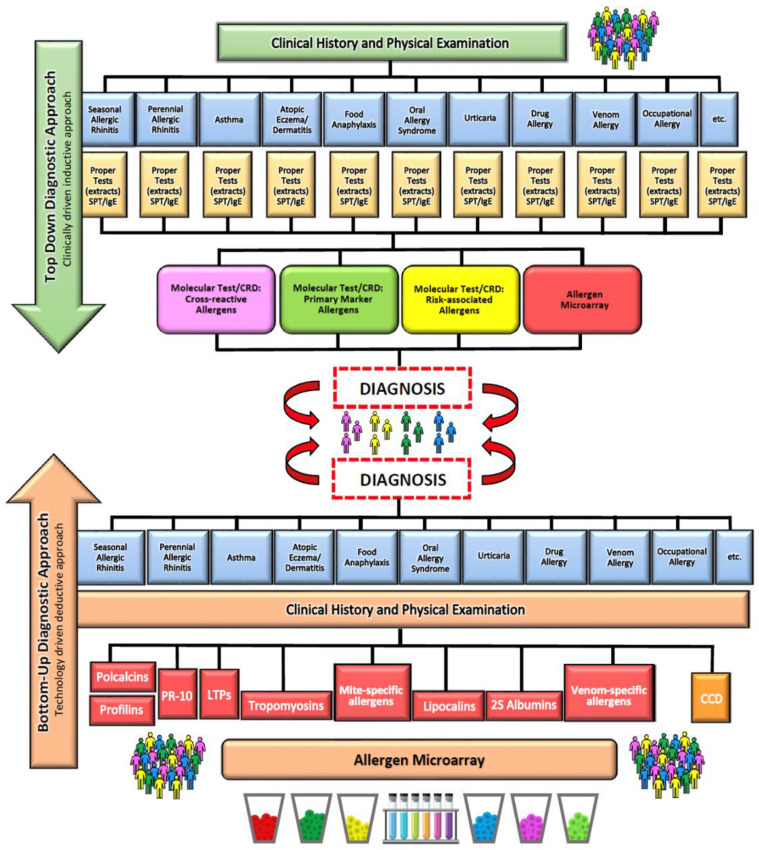
Models used for food allergy diagnosis based on CRD tools [76].

sIgE levels against allergen components can now be measured with blood tests in either singleplex or multiplex systems [78]. In the singleplex system, only one component is tested, and the method is similar to that used for measuring sIgE levels in the whole allergen extract. ImmunoCAP, CAP, radioallergosorbent test assay (RAST), sIgE, and in vitro tests are other terms for this laboratory technique. Multiplex systems measure the concentration of sIgE against more than one allergenic component. Currently, there are two platforms for molecular allergy diagnostics: ISAC introduced in 2006 and ALEX launched in 2016 [75]. Both tools have considerable diagnostic utility when correlated with clinical history [75]. There are two main models for diagnosing allergies that use CRD tools [79]. In a standard approach, the basic tool for allergy diagnosis is a medical interview with reference to clinical symptoms. The next step is in vitro or in vivo tests involving allergen extracts and finally diagnostics based on allergenic molecules. According to the bottom-up model, molecular methods are used at the very beginning of allergy diagnosis, followed by the in vivo or in vitro measurement of sIgE levels against the extracts. The obtained results are then interpreted and compared with the symptoms (Figure 3). The introduction of CRD was undoubtedly a milestone in food allergy diagnosis, as it allowed for better identification and characterization of specific allergenic molecules [79]. However, the sensitivity and specificity of the commercial multiplex tests are too low for CRD to fully replace clinical observation and the DBPCFC.

Identification of allergenic IgE epitopes is essential for the development of new diagnostic and prognostic methods in FA. More accurate IgE profiling, i.e., assessment of antibodies specific for epitopes found on allergen component proteins, could improve diagnostic and prognostic methods by further elimination of irrelevant signals produced by cross-reacting and less specific antibodies. Bead-based epitope assay (BBEA) is a sensitive and reliable tool for determining the profile of epitope-specific antibodies in FA [80]. The high-throughput, highly reproducible bead-based epitope assay is a multiplex test capable of evaluating IgE and IgG4 for over 90 peptide sequences representing various sequential epitopes. Its undeniable advantages are the very low (microliter) amounts of serum or plasma required for the assay, the ease of execution and repeatability, greater accuracy, and the potential to predict clinical outcomes [80]. To date, most allergenic epitope profiles have focused on milk and peanut allergy [78,79,80]. While this technology appears to be very promising, it requires knowledge of the amino acid sequences of the epitopes of allergen proteins contained in each type of food, large cohorts of well-characterized food-allergic patients to validate the tests, and extensive computational skills to develop and validate diagnostic and prognostic algorithms for each food allergen [81]. BBEA, by multiplexing epitopes and processing multiple samples, enables the completion of large experiments in a short time [82]. Peptides, uniquely bound to the beads, are incubated with serum or plasma samples, and after the addition of a fluorophore-labeled secondary antibody, the level of fluorescence is quantified using a Luminex reader. The signal is then normalized and converted into epitope-specific-antibody-binding values [80]. The binding of epitope-specific antibodies, quantified by BBEA, is highly reliable, reproducible, and has greater epitope detection sensitivity than peptide microarrays. In addition, IgE targeting allergenic epitopes can be used to predict the severity of reactions and to identify FA phenotypes [80].

## 7. Diagnosis of Parasitoses

Parasitological diagnostics is a complex area of laboratory medicine involving macroscopic, microscopic, immunological, and molecular methods and sometimes in vitro cultures. The diagnostic method depends on the material and parasite species. The most commonly used techniques are manual ones, and their sensitivity and specificity are limited [80,81,82]. The analyses are mainly based on light microscopy supplemented by coproscopic techniques. The most commonly used methods are flotation or sedimentation [83]. After feces pretreatment and macroscopic inspection, a direct smear is prepared. The next stage is microscopic examination of the material either on a slide (Kato–Katz technique) or, much less often, in a counting chamber (e.g., McMaster) [84]. Standard techniques for detecting parasitosis are time- and labor-consuming. They require extensive experience of the diagnostician and may be inaccurate, as it is very difficult to distinguish some species. Table 1 presents the methods most commonly used in medical laboratories to diagnose parasitic infections.

Reliability of the manual diagnostic methods depends on many factors that may limit their accuracy and diagnostic value. The most important limitations include methodological shortcomings, a small number and uneven distribution of eggs in the feces sample, the reproductive potential of the parasite, irregular egg excretion, and variable infestation frequency in specific geographical regions [82,83]. Studies indicate low diagnostic sensitivity of the methods based on direct microscopy (43–52%), depending on the parasite species [85]. To increase the probability of detecting eggs in the sample, the material may be compacted, but the results of this approach are not satisfactory. For parasites of high reproductive potential, such as *Ancylostoma* or *Haemonchus* spp., the infestation intensity is assessed based on the number of eggs in 1 g of feces [85]. Those solutions are justified in endemic areas or in places with a high frequency of infestation with these species [85].

A reliable method of parasitological diagnosis is immunodiagnostic ELISA tests used for detecting parasite antigens in the feces (coproantigens). Serological tests play a fundamental role in diagnosing diseases caused by tissue parasites. However, not all tests provide high sensitivity and specificity. The results of ELISA tests show a strong correlation between the level of the coproantigen and copro-DNA in the feces of people infected with *Ascaris lumbricoides*, while the correlations for the infections diagnosed with microscopic observations and real-time quantitative PCR (qPCR) are moderate for *Ancylostoma* and strong for *A. lumbricoides* and *T. trichiura* [86]. The accuracy of ELISA tests detecting the coproantigen may be affected by the components found in the fecal samples, such as salts, proteases, antibodies, and organic compounds. The results depend also on the conditions of the sample preservation and storage, as alterations at any of these steps could interfere with antigen detection and produce false-positive or false-negative results [86].

Modern science has high hopes for methods based on artificial intelligence supporting laboratory work. The usefulness of the latest technological developments in the diagnosis of parasitic infections in humans will depend on the outcomes of large-scale studies and the cost–benefit ratio.

The rapid development of sequencing and enzymatic DNA duplication (PCR) techniques has allowed for supplementing parasitology diagnostics with molecular methods based on nucleic acid analysis. Until recently, they were mainly used in academic laboratories, and large-scale experiments were limited [87]. The advantage of genome analysis-based techniques over standard methods is that the DNA of an organism usually does not change during its life cycle [88]. According to the recommendations of parasitological societies, molecular tests can be used for detecting low-intensity infestations too limited to be detected with light microscopy [89]. Due to high analytical sensitivity and specificity, nucleic acid amplification techniques (NAATs) are increasingly often used in the coprodiagnosis of parasitic infections in humans. The NAAT molecular method allows for the identification of helminths in fecal extract samples by binding complementary oligonucleotide primers to nucleic acid strands, followed by the enzymatic amplification of DNA or RNA fragments [90]. NAATs are useful in detecting parasitic infestations at sites of low infection intensity, as they increase the sensitivity of the tests and improve the identification of infections [91].

So far, PCR-based tests have been developed for only a few parasitic species. The PCR method requires specialized laboratory chemicals and equipment, which means that it is not widely used. In recent years, a recombinase polymerase amplification (RPA) system in battery-powered portable biosensors and tools based on loop-mediated isothermal amplification (LAMP) have been developed. This high-speed DNA detection system requires no specialized laboratory equipment and is portable and field-ready. Examples include RPA constructed for *S. haematobium* DNA from genomic urine DNA or LAMP detection of intestinal parasites from nucleic acid extracts from adult parasites [89,90]. These achievements pave the way for diagnostic molecular tests in the form of pocket-size, portable detection chips [92].

**Table 1:** Brief characteristics of the most important techniques for identifying parasite eggs or cysts.

Method Name	Method Description	Ref
DIRECT SMEAR	A small amount of fresh stool is emulsified in one drop of saline on a glass slide. The emulsified stool is then covered with a coverslip to obtain a thin smear preparation in which eggs/larvae or trophozoites of various parasite species are looked for under a light microscope.	[93]
KATO–KATZ TECHNIQUE	A stool sample of approximately 50 mg is placed on a glass slide and then covered with a piece of cellophane soaked in glycerol. Then, the slide is inverted and gently pressed to obtain a thin smear. Glycerol “cleanses” the fecal material. The preparation can be examined under a microscope after 1 to 24 h. Eggs detected in the sample are counted and expressed per gram of feces.	[94]
FORMOL-ETHER CONCENTRATION TECHNIQUE	The stool sample is emulsified in water with formol. The resulting suspension is strained to remove large, redundant fecal particles. Then, ether or ethyl acetate is added, and the resulting suspension is centrifuged. Parasitic forms, i.e., cysts, oocysts, eggs, or larvae, are fixed and sedimented, while the fecal remains are suspended in the layer between ether and formol water. The sediment is examined under a light microscope to detect and count the forms of the parasite.	[95]
AGAR PLATE CULTURE	The technique requires agar medium (1.5% agar, 0.5% bovine extract, 1.0% peptone, and 0.5% NaCl). The medium (10 mL) is transferred to a Petri dish and left to cool at room temperature. Then, 2 g of fresh feces is placed in the center of the agar plate and incubated at 26–33 °C. The plates are inspected for characteristic patterns of larval movement every 24 h for up to 72 h. The presence of motile larvae indicates a positive test result.	[96]
ZINC SULFATE FLOTATION	First, 1 g of feces is emulsified in water and strained to eliminate unnecessary residues. The filtrate is centrifuged, and the precipitate is suspended in 4 mL of ZnSO_4_ solution (density: 1.180–1.200). The suspension is left for 30–45 min for the eggs and cysts to float to the surface. A coverslip is placed on top of the test tube to collect parasite eggs/larvae, which are subsequently transferred to a glass slide to verify their presence under the microscope.	[97]
FLOTAC TECHNIQUE	An accurately weighed sample of 1 g or more of feces is collected after thorough homogenization of a large amount of fecal material in water. The homogenized suspension is filtered through a wire mesh into a tube; then, the tube is centrifuged for 3 min at 1500× *g* rpm. The obtained supernatant is discarded, and the selected flotation solution is added to the test tube again. The sample is homogenized to form a suspension that can fill the 2 flotation chambers of the FLOTAC apparatus. The FLOTAC apparatus is then closed, and the samples are centrifuged again for 5 min at 1000× *g* rpm. After centrifugation and transfer of the upper parts of the flotation chambers, they can be read under a microscope.	[98]
MCMASTER EGG COUNTING TECHNIQUE	Two grams of feces is transferred to a beaker containing 60 mL of ZnSO_4_ solution. To homogenize the feces, the resulting suspension is filtered through a gauze or wire mesh into another container. The filtrate is placed in a clean 15 mL tube. A coverslip is placed on its top, and it is left for 15 min. Then, the coverslip is carefully transferred to a slide and examined under a microscope (10× magnification). The suspension is re-homogenized, and both chambers of the McMaster slide are filled using a pipette. The chambers are fixed for up to 3 min so that the eggs float to the top. Debris sediments to the bottom of the chamber. Under the microscope (10×), parasite eggs located in the grid area defined on both sides of the chamber are counted. The number of eggs detected is multiplied by 100 to get the number of eggs per gram of feces.	[96]
POLYMERASE CHAIN REACTION (PCR)	The multiplex qPCR method enables the quantification and detection of several target DNA sequences simultaneously. DNA amplification occurs in real time, and a combination of multiple primer sets is used. Studies with species-specific primers/probes have shown increased detection sensitivity for up to eight gastrointestinal parasitic pathogens.	[99]

## 8. Allergy to Parasites and Cross-Reactivity

Cross-reactivity between parasite antigens, such as tropomyosin, and food allergens may explain the convergence of symptoms of a parasitic disease and allergy [100]. Traveling from places of low infection intensity to areas endemic for specific parasitosis may contribute to a strong inflammatory allergic response to parasites [101]. If it is impossible to quickly eliminate the parasite from the human body, there is a constant release of histamine. This makes the immune system hypersensitive and triggers an allergic response even to substances that have not previously been allergenic [102].

The life cycles of some parasites, including *A. lumbricoides, S. stercoralis*, or *A. duodenale*, include the migration of the larvae through the lungs. In infected individuals, their presence may cause allergic symptoms, such as shortness of breath or wheezing [103]. A study from the Republic of South Africa involving children infested with *A. lumbricoides* showed an increased probability of detecting a marker of airway hyperresponsiveness [104]. Similarly, a study of 2316 people from the fishing communities of Lake Victoria in Uganda revealed a positive correlation between *A. lumbricoides* infection and allergic symptoms [105].

In the late 1960s, one of the first studies linking the then newly characterized IgE with parasitosis was published [106]. Ethiopian preschoolers infected with *A. lumbricoides* showed an up to 20 times higher concentration of total IgE than the uninfected children [106]. Even the early study by Johansson et al. indicated parasitic infestation as an important factor stimulating the synthesis of IgE antibodies in children with parasitosis [106]. Subsequently, several studies from sub-Saharan Africa reported elevated levels of allergen-specific IgE associated with parasitic infection, but these did not translate into allergic symptoms [97,104]. This phenomenon can be associated with IgE cross-reactivity, where immunoglobulins targeting one epitope can identify similar epitopes in homologous molecules [105,106,107].

In allergy research, the protein that evokes the initial allergic response is referred to as the “primary sensitizer”, and consecutive molecules are considered cross-reacting allergens [108]. Two types of allergy-linked cross-reactivity have been described in the literature. The first is mediated by proteins and the second by carbohydrate structures on glycoproteins, known as cross-reactive carbohydrate determinants (CCDs) [109].

A study conducted in Ghana among 1604 school children demonstrated the role of parasites in carbohydrate cross-reactivity. The children were tested for *Schistosoma haematobium* infection and peanut allergy. Those with an increased IgE concentration upon exposure to peanut extract did not show any allergic symptoms [110]. A total of 18% of children were allergic to peanuts (Immunocap ≥ 0.35 kU/L), but 92% were PT negative [100]. *S. haematobium* infection positively correlated with peanut allergy. Moreover, a strong correlation was observed between IgE anti-CCD and IgE specific to whole peanut extract [110]. Inhibition tests in a subgroup of children showed that a soluble antigen of *S. haematobium* egg and CCD marker bromelain strongly inhibited IgE binding to peanut extract. CCD-targeted IgE impedes the in vitro diagnosis of allergen-specific IgE by yielding false-positive results, and blocking CCD-targeted IgE improves the detection of allergen-specific IgE [110]. A CRD analysis of Ghanaian children with elevated IgE upon exposure to peanut extract, which was associated with *S. haematobium* infection, revealed a very weak response to recombinant peanut allergens Ara h 1, 2, and 3 as compared with the extract [110]. Wollmann et al. used a CRD to assess IgE reactivity to peanut allergen components (Ara h 1–3, 6, 8, 9) among allergic patients in Zimbabwe who were allergic to whole peanut extract but had no allergic symptoms [111]. A total of 46% of the patients responded with elevated IgE to at least one of the highly allergenic components of peanuts. However, 50% of the patients had elevated IgE against CCD [111]. The studies referenced above indicate CRD as a useful tool in improving the specificity of in vitro diagnostics of parasitosis-associated allergy.

## 9. Tropomyosin—A Common Allergen Found in Animal Foods and Parasites

The main cause of an allergy to crustaceans is tropomyosins (TMs), belonging to the family of muscle-building proteins. They are strongly allergenic and resistant to high temperatures. Tropomyosin is a panallergen responsible for molecular and clinical cross-reactivity between crustaceans and mollusks and homologs of mites, cockroaches, and some nematodes, such as *Ascaris* [112].

The amino acid sequence of tropomyosins is highly conserved throughout the animal kingdom. TM sequence similarity among different invertebrate species reaches over 75–80%, which is the reason for a large number of cross-reactions among allergic individuals [112]. A food challenge study by Broekman et al. showed that of 13 patients allergic to shrimps, 11 were also allergic to mealworm [113]. Further tests confirmed cross-reactivity with tropomyosin. Table 2 compares tropomyosins present in the parasite *Anisakis simplex*, the mite *Dermatophagoides pteronyssinus*, and shrimps.

*A. lumbricoides* tropomyosin (Asc l 3) has strong allergenic properties [114]. In tropical regions, Asc l 3 is associated with the occurrence and severity of asthma, which may indicate its clinical importance [115]. Due to its cross-reactivity with mite tropomyosins, this protein may exacerbate dust mite allergy. The so far characterized allergenic antigens of *A. lumbricoides* include polyprotein As s 1 (ABA-1) and glutathione transferase As 1 13 [116]. Other parasite allergenic proteins include *Anisakis simplex* Ani s 1, Ani s 4, Ani s 7, and Ani s 9, *Necator americanus* NaASP2q and Nacal1, and *Schistosoma mansoni* SmVAL4 and Sm22.6 [111,113].

## 10. Anisakis—A Parasite Triggering IgE-Mediated Host Response

*Anisakis simplex* is a parasitic nematode common throughout the world. The source of infection for humans is undercooked or raw seafood contaminated with the parasite. The vast majority of cases concern the inhabitants of Spain and Japan. In recent years, there has been a significant increase in the number of publications on *Anisakis* allergy in many different regions of the world [115,116].

*A. simplex* causes IgE-mediated hypersensitivity reactions [117]. It is the most studied nematode with the largest number of known allergens [118]. The allergenic proteins of *A. simplex* can be divided into two groups: excretory–secretory antigens produced during the excretion of larvae from the host alimentary tract or during a surgical intervention and somatic antigens from the alive and dead larvae of the parasite [119].

So far, 14 allergens of *Anisakis simplex* have been characterized. Six of them are considered thermostable, which means that immunologic response in the form of an allergic reaction can occur after the digestion of even dead larvae of *Anisakis simplex* [117,118,119]. These allergens include Ani s 1, Ani s 4, Ani s 5, Ani s 8, Ani s 9, and Ani s 10. Some allergens have specific characteristics; for example, Ani s 4 and Ani s 6 are resistant to gastric pepsin [120]. The proteins that evoke allergies in over 50% of patients are called major allergens, and they include Ani s 1, Ani s 2, Ani s 7, Ani s 12, Ani s 13, and Ani s 14 [121,122]. Ani s 2 and Ani s 3 are so-called panallergens, and they are responsible for cross-reactions with other food allergens [123].

Below, we present the allergens isolated from *A. simplex:*

− Ani s 1 (24 kDa): the main, thermostable allergen present in many isoforms. It is a serine protease inhibitor similar to serine protease inhibitors from *Caenorhabditis elegans* [124]. It is released by secretory glands and is detected in 86% of patients with IgE-mediated allergy to Ani s 1 [125].
− Ani s 2 (100 kDa): paramyosin detected in 88% of people with an allergic reaction.
− Ani s 3 (41 kDa): tropomyosin present in the larval muscles, a somatic antigen playing a crucial role in cross-reactivity due to considerable homology with myosines of other organisms, detected in about 13% of patients with allergic symptoms [126]. Some researchers underline the importance of Ani s 3 as an allergen present in food [127].
− Ani s 4 (9 kDa): thermostable inhibitor of cysteine proteinase, secreted by the excretory glands and present in the basal layer of the epidermis in the third-stage larvae [116]. There are two isoforms of Ani s 4 containing either leucine or proline at the third position of the mature protein. The leucine-containing isoform was shown to be more allergenic than the proline-containing one. Moreover, the first isoform plays an important role as a trigger of anaphylactic reactions [116]. As many as 27% of allergic people respond to Ani s 4 [128].
− Ani s 5 (15 kDa): a weak, thermostable allergen responsible for cross-reactivity, produced by the secretory glands, stomach, and luminal surface of the larval intestinal epithelium. A response to this allergen was confirmed in 49% of people allergic to *Anisakis simplex* [129].
− Ani s 6 (7 kDa): a serine protease inhibitor showing considerable homology with other serine protease inhibitors, including those from *Boophilus microplus*, *Anopheles stephensi*, *Glossina morsitans*, and *Apis mellifera* [116]. A reaction to the recombinant rAni s 6 allergen was confirmed in 18% of patients [130].
− Ani s 7 (139 kDa): a serine protease inhibitor. A reaction to this allergen was detected in 83–100% of people allergic to *Anisakis simplex* [131]. A high titer of sIgE antibodies against Ani s 7 is an indicator of a new infection.
− Ani s 5 (15 kDa), Ani s 8 (15 kDa), and Ani s 9 (14 kDa) are members of the SPX/RAL-2 family sharing amino acid sequence homology. Ani s 9 is structurally similar to the antigens of *Ascaris suum* or *Acanthocheilonema viteae* [132]. Cross-reactivity has been reported between Ani s 9 and wasp venom allergens. A response to Ani s 8 was confirmed in 25% of allergic patients, while a response to Ani s 9 was confirmed in 13.8% of allergic individuals [133,134].
− Ani s 10 (22 kDa): probably a somatic antigen of unknown function. It has seven amino acid repeats, each expressing a cleavage site for trypsin and pepsin. It is putatively cleaved in the alimentary tract into seven active peptides [122]. An allergy to it was diagnosed in 39% of tested patients [122].
− Ani s 11 (55 kDa) and Ani s 12 (31 kDa): proteins of unknown function that were identified by the chemiluminescence screening of the *Anisakis simplex* cDNA expression library. The proteins were described by Kobayashi et al., who reported that Ani s 11 has five or six short repetitive sequences containing from 6 to 15 amino acids, and Ani s 12 has a tandem motif with four cysteine residues [124]. According to the researchers, the reaction to Ani s 11 and Ani s 12 appears in about 50% of people [124].
− Ani s 13 (37 kDa): *A. simplex* hemoglobin considered one of the major allergens [133]. It belongs to a conserved family of proteins and is not responsible for cross-reactivity [135].
− Ani s 14 (24 kDa): a protein of unknown function, characterized by two homologous regions similar to the sequences found in Ani s 7 and Ani s 12 allergens and redundant sequences, probably acting as an IgE binding site [135]. It was detected in 54% of allergic patients [136].

## 11. Conclusions

The analysis of the course of the host’s immune system response to allergens and parasite infections reveals a number of similarities. They include symptoms present at an early stage of FA and parasitosis development. Moreover, some parasites are highly allergenic to humans. All these factors make it difficult for a physician to establish an accurate diagnosis. Enriching and extending the algorithm of allergological diagnostics with parasitology tests seems justified, as it allows for shortening the diagnosis time, lowering its costs, and the quick beginning of an effective therapy.

## Figures and Tables

**Table 2:** Allergenic tropomyosin from *Anisakis simplex*, *Dermatophagoides pteronyssinus*, and brown shrimp [79].

Allergen Characteristics				
Biochemical Name		Tropomyosin		
Allergen source	Major taxonomic group	Animalia *Nematoda*	Animalia *Arthropoda*	Animalia *Arthropoda*
	Order	*Ascaridida*	*Astigmata*	*Decapoda*
	Species	*Anisakis simplex*	*Dermatophagoides pteronyssinus*	*Penaeus aztecus* (brown shrimp)
Allergen name		Ani s 3	Der p 10	Pen a 1
Biological function		Tropomyosin, together with the troponin complex, plays a central role in the calcium-dependent regulation of muscle contraction.		
Structural model predicted by AlphaFold		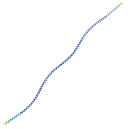	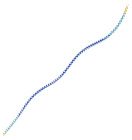	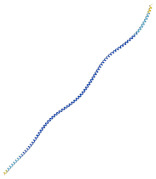
Molecular weight		33,203 Da	32,553 Da	32,849 Da
Length		284 aa	281 aa	284 aa
Route of allergen exposure		food	airway	food
UniProt		Q9NAS5	Q304Y3	Q3Y8M6

## Data Availability

No new data were created or analyzed in this study. Data sharing is not applicable to this article.
